# Identification of four genes associated with cutaneous metastatic melanoma

**DOI:** 10.1515/med-2020-0190

**Published:** 2020-06-11

**Authors:** Chen Ji, Yuming Li, Kai Yang, Yanwei Gao, Yan Sha, Dong Xiao, Xiaohong Liang, Zhongqin Cheng

**Affiliations:** Department of Dermatology, Zhangjiagang TCM Hospital Affiliated to Nanjing University of Chinese Medicine, 77 Changan South Road, Zhangjiagang, Jiangsu Province, 215600, China; Department of Pulmonary and Critical Care Medicine, Zhangjiagang TCM Hospital Affiliated to Nanjing University of Chinese Medicine, 77 Changan South Road, Zhangjiagang, Jiangsu Province, 215600, China

**Keywords:** cutaneous melanoma, metastasis, prognosis, gene analysis

## Abstract

**Background:**

Cutaneous melanoma is an aggressive cancer with increasing incidence and mortality rates worldwide. Metastasis is one of the primary elements that influence the prognosis of patients with cutaneous melanoma. This study aims to clarify the potential mechanism underlying the low survival rate of metastatic melanoma and to search for novel target genes to improve the survival rate of patients with metastatic tumors.

**Methods:**

Gene expression dataset and clinical data were downloaded from The Cancer Genome Atlas portal. Differentially expressed genes (DEGs) were identified, and their functions were studied through gene ontology and Kyoto Encyclopedia of Genes and Genomes enrichment analyses. Survival and multivariate Cox regression analyses were used to screen out candidate genes that could affect the prognosis of patients with metastatic melanoma.

**Results:**

After a series of comprehensive statistical analysis, 464 DEGs were identified between primary tumor tissues and metastatic tissues. Survival and multivariate Cox regression analyses revealed four vital genes, namely, *POU2AF1*, *ITGAL*, *CXCR2P1*, and *MZB1*, that affect the prognosis of patients with metastatic melanoma.

**Conclusion:**

This study provides a new direction for studying the pathogenesis of metastatic melanoma. The genes related to cutaneous metastatic melanoma that affect the overall survival time of patients were identified.

## 1 Introduction

Cutaneous melanoma is a highly malignant and invasive skin cancer, in which melanocytes switch to cancerous cells through variations at molecular and biochemical levels [1]. The incidence rates of melanoma continuously increase [2]. The 5-year overall survival rate for patients in all stages is 92%, whereas that of patients with advanced metastasis (Stage IV) is 23% [2]. Patients with metastatic melanoma tend to have a poor prognosis. Although various treatments, including surgery, chemotherapy, and radiotherapy, are often effective, no certain treatment can improve the overall survival rate due to the consequence of recurrence and severe metastasis relevant to cutaneous melanoma [3]. Thus, potential biomarkers that can evaluate the prognosis of patients with metastatic melanoma and that can serve as potential therapeutic targets for these patients must be explored.

This study aimed to identify the differentially expressed genes (DEGs) between primary and metastatic melanoma and to determine their main functions through a series of comprehensive biostatistical analyses by using the data from The Cancer Genome Atlas (TCGA) public database. Candidate genes that affect the prognosis of patients with metastatic melanoma were further identified by survival and multivariate cox regression analyses.

## 2 Methods

### 2.1 Data sources

Gene expression chart and clinical information were obtained from the TCGA portal as of 17 September 2019. All 472 cutaneous melanoma samples were matched with the corresponding dataset of clinical information for this study. Some samples with no records of the clinical data (*n* = 4), survival data (*n* = 20), and solid normal tissue (*n* = 1) were excluded. In total, 447 samples, including 354 cutaneous metastatic melanoma and 93 primary tumors, were included. Ethics approval was not needed because TCGA is an available public database.

### 2.2 Differential gene expression in cutaneous melanoma

DEGs were determined by using the edgeR package, and *P* < 0.01 and |log FC| ≥ 1 were considered statistically significant. Volcano maps were applied to illustrate the results clearly by R package. Cluster profile R package was used to conduct the gene ontology (GO) analysis with a cutoff of *P* < 0.01 and Kyoto Encyclopedia of Genes and Genomes (KEGG) pathway analysis with a cutoff of *P* < 0.05 and to clarify the biological functional category of these DEGs. Search Tool for the Retrieval of Interacting Genes/Proteins (STRING) database was used to construct the interaction network of DEGs with regard to the connection between these genes. Cytoscape software was used to reveal the interaction between the DEGs. Finally, the crucial genes in the interaction network were determined according to the number of edges connected by each gene.

### 2.3 Survival analysis to select the candidate genes

Univariate Cox regression analysis was applied to evaluate the effect on the expression of these DEGs on overall survival time. Multivariate cox regression analysis was then performed on the top 30 genes according to the results of the univariate Cox regression analysis to distinguish the candidate genes, which are the independent risk factors of prognosis for patients with cutaneous metastatic melanoma.

### 2.4 Analysis of clinical features

Based on the univariate Cox regression analysis, the gene with the biggest impact on the overall survival time of patients with cutaneous metastatic melanoma was selected for further analysis to investigate the correlation between the expression level of a candidate gene and clinical characteristics. The samples were divided into high expression and low expression groups according to the expression level of the genes, and the Chi-square test was then conducted. Clinical characteristics such as age, gender, TNM stage, and tumor status obtained from the TCGA dataset were included.

## 3 Results

### 3.1 DEGs in cutaneous metastatic melanoma

**Figure 1:**
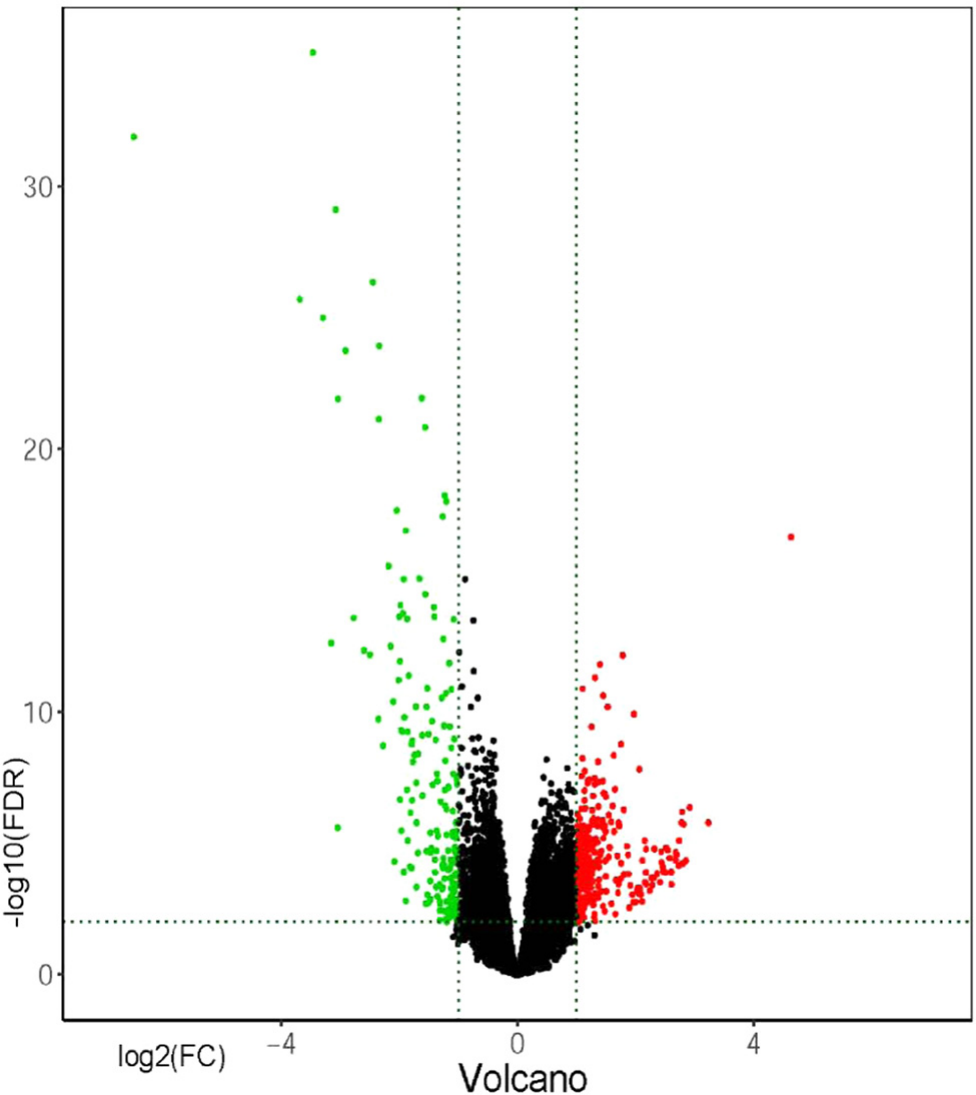
The DEGs of metastasizes and primary tumors. The *y*-axis value is log FC, and the *x*-axis value is −log 10(FDR). The red plots represent the upregulated DEGs, while green plots represent the downregulated DEGs.

**Figure 2:**
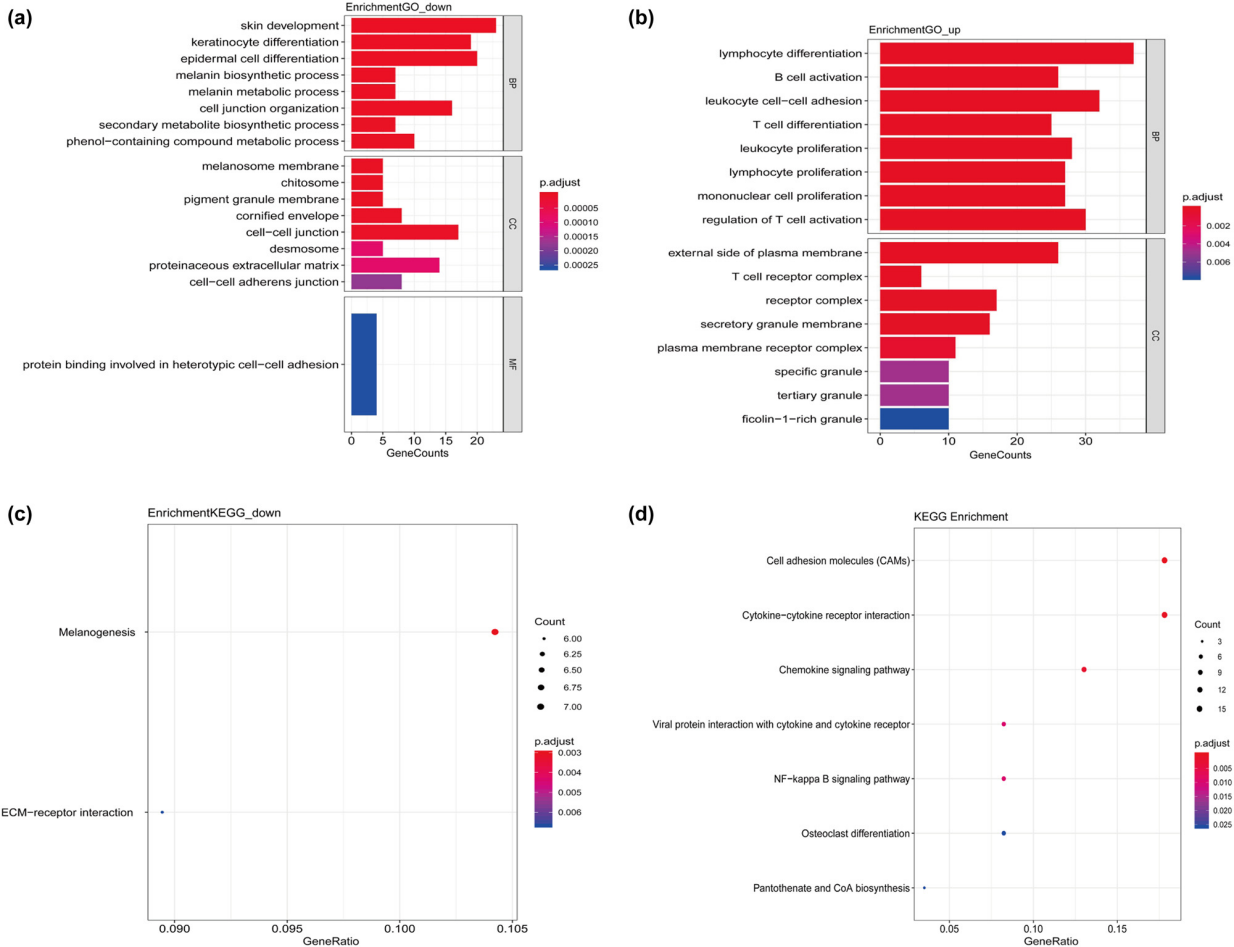
GO and KEGG enrichment analyses of differentially expressed genes. (a) GO analysis results of the downregulated DEGs. (b) GO analysis results of the upregulated DEGs. (c) KEGG analysis results of the downregulated DEGs. (d) KEGG analysis results of the upregulated DEGs.

**Figure 3:**
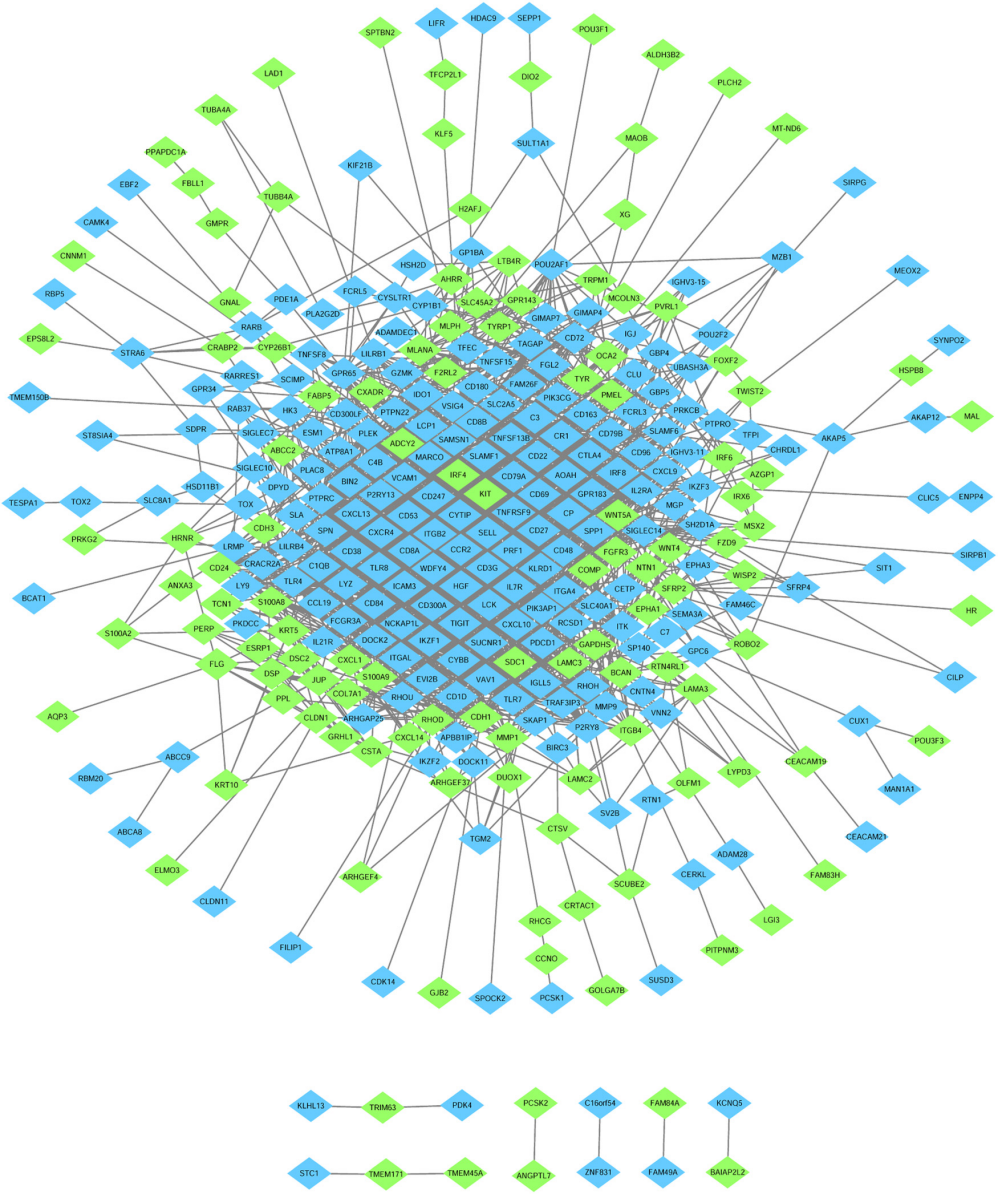
The protein–protein interaction network of DEGs. The nodes represent proteins that correspond to the DEGs. Upregulated DEGs are represented in blue, while downregulated DEGs are represented in green. The proteins connected by edges interact with each other.

**Figure 4:**
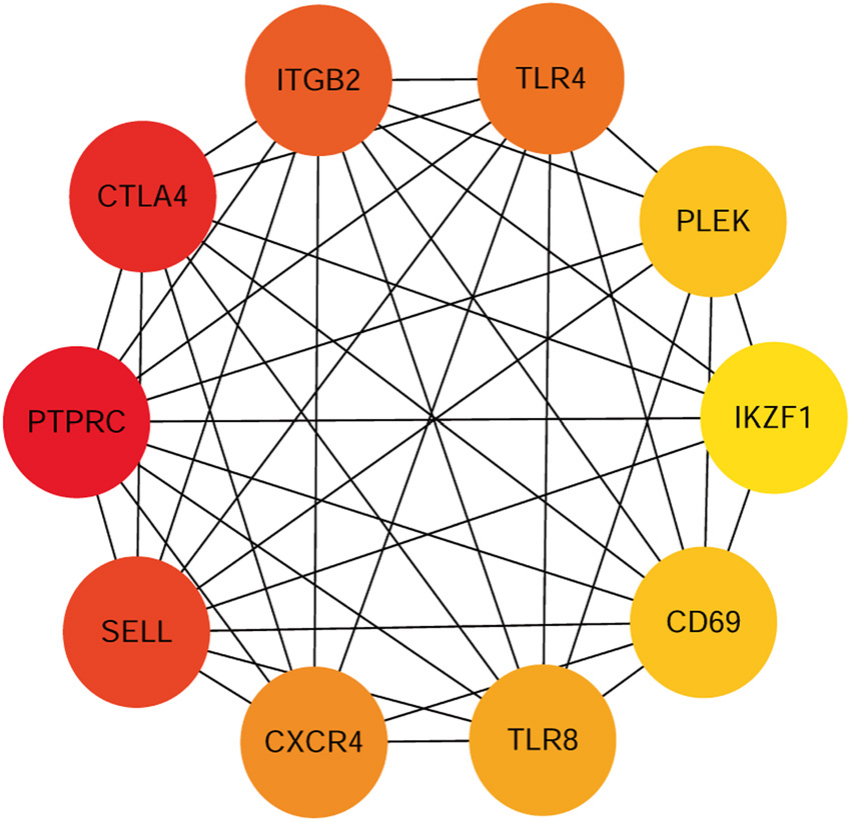
Hub genes in protein–protein interaction network. The top10 hub genes ranked by the degree in the protein–protein interaction network make up a subnetwork.

After data screening and processing by R software, standardized data were used to compare the gene expression in primary and metastatic tumors. The edgeR package identified 464 DEGs between primary tumor tissues and cutaneous metastasis tissues. Among these DEGs, 177 were downregulated and 287 were upregulated according to the cutoff of *P* < 0.01 and |log FC| > 1. Volcano map was used to illustrate the results (Figure 1). GO and KEGG analyses were performed to investigate the functions in which these DEGs were enriched (Figure 2). Downregulated DEGs were enriched in the skin development, keratinocyte differentiation, epidermal cell differentiation, melanin biosynthetic process, and melanin metabolic process. Meanwhile, upregulated DEGs were mainly enriched in lymphocyte differentiation, B-cell activation, leukocyte cell-to-cell adhesion, T cell differentiation, and leukocyte proliferation. KEGG pathway enrichment was also performed to identify the enriched pathways of these genes. The signaling pathways primarily enriched by the downregulated DEGs were the ECM–receptor interaction and tyrosine metabolism signaling way, whereas those primarily enriched in upregulated DEGs were cell adhesion molecules (CAMs), cytokine-to-cytokine receptor interaction, chemokine signaling pathway, viral protein interaction with cytokine and cytokine receptor, and NF-kappa B signaling pathway. STRING (a database of known and predicted protein interactions) was used to predict protein interactions among these DEGs. We then constructed a protein–protein interaction (PPI) network of DEGs, and Cytoscape software was applied to construct a network visualizing the result with 338 nodes and 2,061 edges (Figure 3). The nodes represent the proteins that correspond to the DEGs, and the proteins connected by edges interact with each other. Among them, PTPRC, CTLA4, SELL, ITGB2, TLR4, CXCR4, TLR8, PLEK, CD69, and IKZF1 with high degrees are considered as hub-genes, which suggests they may play important roles in the development of metastasis of melanoma (Figure 4).

### 3.2 Survival analysis

Cutaneous melanoma is a highly invasive disease, and the appearance of metastatic tumor indicates its further deterioration. The survival analysis indicated that 147 DEGs have a significant effect on the overall survival time with a cutoff of *P* < 0.01. The top 30 DEGs, including *IGLV2-14*, *MZB1*, *CD27*, *POU2AF1*, *CD72*, *PDCD1*, *HSH2D*, *PRF1*, *SIT1*, *LY9*, *PLA2G2D*, *IGLV2-8*, *RNF39*, *CXCL9*, *CD38*, *CXCR2P1*, *SIRPG*, *IGKV4-1, FCRL5*, *RAB37*, *ADAMDEC1*, *LCK*, *IGLV3*-*21*, *IGHV3*-*15*, *IGHM*, *IGLC2*, *ITGAL*, *RHOH*, *IGLC3*, and *ICAM3*, were chosen as the potential risk factors for the prognosis of patients with metastatic tumors (Table 1). In addition, the multivariate Cox regression analysis was performed on these genes to determine the candidate genes that can exhibit a significant prognostic value. Finally, POU2AF1 (*P* = 0.00660, HR = 1.36, *B* = 0.31, CI [1.09–1.70]), ITGAL (*P* = 0.02181, HR = 1.72, *B* = 0.54, CI [1.08–2.75]), CXCR2P1 (*P* = 0.02379, HR = 1.19, *B* = 0.17, CI [1.02–1.37]), and MZB1 (*P* = 0.03646, HR = 1.30, *B* = 0.26, CI [1.02–1.66]) were discovered to be the independent risk factors with *P* < 0.05 and hazard rate (HR) > 1 (Table 2).

**Table 1:** Top30 genes significantly affect the overall survival time of patients by survival analysis

	Symbol	HR	Lower 95	Upper 95	*P*-value
ENSG00000211666	IGLV2-14	1.064586836	1.033802118	1.096288266	2.91 × 10^−5^
ENSG00000170476	MZB1	1.070874267	1.036688679	1.106187151	3.52 × 10^−5^
ENSG00000139193	CD27	1.104194794	1.053468406	1.157363747	3.62 × 10^−5^
ENSG00000110777	POU2AF1	1.076582536	1.039061559	1.115458411	4.56 × 10^−5^
ENSG00000137101	CD72	1.142400427	1.07114116	1.218400324	5.09 × 10^−5^
ENSG00000188389	PDCD1	1.088919949	1.044023795	1.135746773	7.33 × 10^−5^
ENSG00000196684	HSH2D	1.093259058	1.046041572	1.142607904	7.55 × 10^−5^
ENSG00000180644	PRF1	1.100839397	1.049524241	1.154663542	7.99 × 10^−5^
ENSG00000137078	SIT1	1.097245862	1.047646805	1.149193103	8.42 × 10^−5^
ENSG00000122224	LY9	1.09801634	1.047826183	1.150610572	8.97 × 10^−5^
ENSG00000117215	PLA2G2D	1.062038864	1.029489807	1.095617015	0.000150662
ENSG00000278196	IGLV2-8	1.060655117	1.028816517	1.09347902	0.000152534
ENSG00000204618	RNF39	0.858613102	0.792757339	0.92993962	0.00018116
ENSG00000138755	CXCL9	1.075199152	1.035120947	1.116829121	0.000183342
ENSG00000004468	CD38	1.092412444	1.042907907	1.144266851	0.000187308
ENSG00000229754	CXCR2P1	1.069877224	1.032359785	1.108758101	0.000208432
ENSG00000089012	SIRPG	1.083532648	1.038542691	1.130471581	0.000209063
ENSG00000211598	IGKV4-1	1.055884647	1.025779669	1.086873157	0.000229057
ENSG00000143297	FCRL5	1.062535038	1.028736881	1.097443602	0.000235294
ENSG00000172794	RAB37	1.105656553	1.047967049	1.166521805	0.000239164
ENSG00000134028	ADAMDEC1	1.075612496	1.03447626	1.118384525	0.000248689
ENSG00000182866	LCK	1.090707281	1.041152223	1.14262098	0.000252373
ENSG00000211662	IGLV3-21	1.055848619	1.025517144	1.087077202	0.000257917
ENSG00000211943	IGHV3-15	1.055896242	1.025467865	1.08722751	0.000266718
ENSG00000211899	IGHM	1.057699963	1.026263913	1.090098947	0.000268378
ENSG00000211677	IGLC2	1.060772	1.027622014	1.094991369	0.000270526
ENSG00000005844	ITGAL	1.098116455	1.044162118	1.154858741	0.000271455
ENSG00000168421	RHOH	1.09005463	1.040546325	1.141918499	0.000277017
ENSG00000211679	IGLC3	1.060198803	1.027263638	1.094189905	0.00028281
ENSG00000076662	ICAM3	1.096557746	1.042964911	1.152904453	0.000311657

**Table 2:** Results of multivariate Cox regression analysis of top30 genes

	Symbol	Coef.	HR	Lower 95	Upper 95	Pr (>|*z*|)
ENSG00000211666	IGLV2–14	0.019054	1.019237	0.8858	1.1728	0.79017
ENSG00000170476	MZB1	0.262876	1.300665	1.0167	1.6639	0.03646*
ENSG00000139193	CD27	0.103152	1.10866	0.7666	1.6034	0.58371
ENSG00000110777	POU2AF1	0.30893	1.361966	1.0898	1.7021	0.0066*
ENSG00000137101	CD72	−0.276222	0.758644	0.5625	1.0232	0.07033
ENSG00000188389	PDCD1	−0.129255	0.878749	0.6704	1.1519	0.34929
ENSG00000196684	HSH2D	−0.268995	0.764147	0.589	0.9913	0.04279*
ENSG00000180644	PRF1	−0.017232	0.982916	0.7773	1.2429	0.88557
ENSG00000137078	SIT1	−0.186718	0.829678	0.5577	1.2342	0.35679
ENSG00000122224	LY9	−0.086585	0.917057	0.6824	1.2324	0.56587
ENSG00000117215	PLA2G2D	−0.227453	0.79656	0.6802	0.9328	0.00476*
ENSG00000278196	IGLV2–8	0.003758	1.003765	0.8987	1.1211	0.9469
ENSG00000204618	RNF39	0.081054	1.084429	0.9514	1.2361	0.22498
ENSG00000138755	CXCL9	0.024433	1.024733	0.8602	1.2208	0.78441
ENSG00000004468	CD38	−0.248007	0.780355	0.6448	0.9444	0.01084*
ENSG00000229754	CXCR2P1	0.170242	1.185592	1.0229	1.3742	0.02379*
ENSG00000089012	SIRPG	0.085592	1.089361	0.7577	1.5662	0.64404
ENSG00000211598	IGKV4-1	0.068116	1.07049	0.9404	1.2186	0.30297
ENSG00000143297	FCRL5	−0.123312	0.883988	0.6869	1.1376	0.33804
ENSG00000172794	RAB37	0.024174	1.024469	0.8445	1.2428	0.80625
ENSG00000134028	ADAMDEC1	−0.032565	0.96796	0.8507	1.1013	0.62103
ENSG00000182866	LCK	−0.004812	0.9952	0.6297	1.5728	0.98356
ENSG00000211662	IGLV3–21	0.030352	1.030817	0.9201	1.1549	0.60058
ENSG00000211943	IGHV3–15	−0.028086	0.972304	0.8631	1.0954	0.64412
ENSG00000211899	IGHM	0.053087	1.054521	0.9358	1.1882	0.3835
ENSG00000211677	IGLC2	−0.164464	0.848348	0.6886	1.0451	0.12231
ENSG00000005844	ITGAL	0.544648	1.724001	1.0825	2.7457	0.02181*
ENSG00000168421	RHOH	−0.040537	0.960274	0.7026	1.3125	0.7993
ENSG00000211679	IGLC3	−0.088797	0.915031	0.7835	1.0687	0.26214
ENSG00000076662	ICAM3	0.0512	1.052534	0.7176	1.5438	0.79332

Significant codes: 0 “***”, 0.001 “**”, 0.01 “*”, 0.05 “.”, 0.1 “” 1.

### 3.3 Clinicopathological parameters with regard to the expression of *MZB1*

Compared with the other three candidate genes, *MZB1* has the biggest impact on the overall survival time according to the survival analysis. The Chi-square analysis was performed to evaluate the connection between the expression of *MZB1* and clinical characteristics such as gender, age, clinical stage, pathologic-T, pathologic-M, pathologic-N, and neoplasm status. According to the analysis, the expression level of *MZB1* was significantly associated with pathologic-N (*P* = 0.046) and tumor status (*P* = 0.002) in patients with cutaneous melanoma (Table 3). By contrast, characteristics such as clinical stage, gender, age, pathologic-T, and pathologic-M were not connected with the expression of *MZB1* (all *P* > 0.05).

**Table 3:** Clinicopathological parameters of patients with cutaneous melanoma with regard to the expression of MZB1

Characteristic	Expression of MZB1		*P*-value
	Low	High	
Age, years			0.566
<60	46	51	
≥60	176	171	
Gender			0.105
Female	76	93	
Male	147	131	
Tumor_status			0.002^a^
Tumor free	86	123	
With tumor	119	93	
Pathologic-T			0.166
Tis + T1 + T2	60	64	
T3 + T4	129	101	
Pathologic-M			0.505
M0	197	202	
M1	13	10	
Pathologic-N			0.046^a^
N0	120	102	
N1 + N2 + N3	76	97	

^a^ *P* < 0.05. T, tumor; M, metastasis; N, node.

## 4 Discussion

Cutaneous melanoma is an invasive disease with high recurrence and metastasis rates. The overall survival time of patients with cutaneous metastatic tumors was remarkably shorter than that of patients without metastases, suggesting that the presence of metastases always means a poor prognosis. Identifying vital molecules that participate in the pathogenesis of metastatic melanoma is valuable for the potential development of therapeutic targets. Various biomarkers of cutaneous metastatic melanoma have been previously identified. However, the results are always different due to the variations in the samples and the focus of the analysis. Glypican 6 expression is higher in metastatic melanoma than in primary melanoma and is higher in primary melanoma than in normal melanocytes; therefore, this gene may be a biomarker for the metastatic progression of melanoma [4]. Glypican 6 is used to distinguish between the primary tumors of melanoma and regional cutaneous/subcutaneous metastases during early metastasis. The expression of nestin, an intermediate filament that can be a biomarker for stem cells, is also different between primary tumors and cutaneous metastatic melanoma [5]. Dong Wei identified that *ATF2*, *SOX2*, and *RAC1* are involved in the metastasis of melanoma [6]. This article focused on the functional analysis of DEGs and did not explore the impact of DEGs on prognosis.

In our study, 464 DEGs were identified by the edgeR package, in which 177 were downregulated and 287 were upregulated. GO and KEGG enrichment analyses were conducted in these DEGs. The univariate Cox regression analysis was applied for the survival analysis based on the diverse expression of these DEGs. The top 30 DEGs with the most significant influence on the overall survival time according to the *P*-value were selected for further multivariate Cox regression analysis. Four candidate genes, namely, *POU2AF1*, *ITGAL*, *CXCR2P1*, and *MZB1*, were considered to be the substantial independent risk factors for metastatic cutaneous melanoma.

*POU2AF1*, which was upregulated in the cutaneous metastatic melanoma, is a B cell-specific coactivator that can stimulate the gene transcription. Its expression is regulated by the B-cell receptor (BCR) and CD40-L; a continuous stimulation may lead to an overexpression of this gene on B cells [7]. *POU2AF1* plays a role in the pre-B1-to-pre-B2 cell transition and affects the pre-BCR and BCR signaling at multiple stages of B-cell development [8].

*POU2AF1* is closely related to the immune system and various lymphopoietic system diseases. Kan Zhai’s study revealed that the gene mutation in 3′-UTR regulates *POU2AF1* expression and subsequently gives rise to lymphoma [9]. The co-expression of *POU2AF1* and *Oct-2* can be a helpful prognosis for patients with acute myeloid leukemia (AML) [10]. *POU2AF1* helps in the progression of multiple myeloma (MM) when activated by amplification or other mechanisms [11]. In an analysis of gastrointestinal stromal tumors, *POU2AF1* was found to be one of the four genes that act as biomarkers for the prognosis of the high-risk gastrointestinal stromal tumors [12].

*ITGAL*, also known as *CD11a*, is upregulated in metastatic melanoma, highly expressed in most immune cell populations, and encodes a subunit of *LFA-1* integrin [13]. *LFA-1* interacts with its ligand, *ICAMs* 1–3, which acts as a rolling and signaling molecule that plays a crucial role in intercellular adhesion between white blood cells and lymphocyte co-stimulation signaling [14,15]. *LFA-1* also mediates lymphocyte, monocyte, natural killer cell, and granulocyte interaction with other cells in immunity and inflammation [16].

*ITGAL* is closely linked to the pathogenesis of diverse immune-related diseases. In addition, *ITGAL* or *LFA-1* encoded by *ITGAL* plays a role in various tumors. In the research of prostate cancer, the expression of *ITGAL*, along with four other genes, is connected with a number of positive lymph nodes [17]. *ITGAL* is also involved in immune response, inflammatory response, and formation of the tumor microenvironment, thus contributing to the pathogenesis of head and neck squamous cell carcinoma [18]. *ITGAL* is one of the prognostic factors for the survival and the risk of death of men with castration-resistant prostate cancer [19]. This finding is consistent with the results of the present study, in which the high expression of *ITGAL* is a prognostic risk factor for metastatic melanoma. By contrast, *LFA-1* and *ICAM-1* upregulated by IL-18 can facilitate the eosinophil-mediated tumoricidal activity against a colon carcinoma cell line [20].

*MZB1* (or *pERp1*) is upregulated in the metastatic melanoma and is a B cell-specific and endoplasmic reticulum (ER)-localized protein that is abundantly expressed in marginal zone B and B1 cells [21]. *MZB1* regulates calcium signaling, antibody secretion, integrin-mediated adhesion, and lymphocyte adhesion and migration [22,23]. Herold et al. found that the expression of *MZB1* can be a valuable prognostic factor for different lymphoma subtypes [24]. *MZB1* is also a biomarker of favorable prognosis in pancreatic cancer resected after the neoadjuvant chemoradiotherapy [25]. By reviewing the studies, we found that the impact of B lymphocytes on tumors has two sides. On the one hand, B lymphocytes can produce tumor antigen-specific immunoglobulin G antibodies [26]. On the other hand, B lymphocytes scattered in the tumor stroma suppress the antitumor immunity [27]. Hence, we speculated that MZB1 can be a favorable or a poor prognosis in different tumors.

*CXCR2P1* upregulated in the metastatic melanoma is also known as *CXCR2P* and *IL8RBP*. The high expression level of *CXCR2* is always relevant to metastasis and poor prognosis of tumors. *CXCR2* can facilitate breast cancer metastasis and chemoresistance [28] and gastric cancer metastasis [29]. Interleukin-8 promotes cell migration via *CXCR1* and *CXCR2* in liver cancer [30]. In malignant melanoma, the expression of *CXCL8* and *CXCR2* promotes aggressive growth and metastasis [31]. Singh et al. found that the host’s *CXCR2* contributes to the melanoma growth, angiogenesis, and experimental lung metastasis in mice [32]. Small molecule antagonists targeting *CXCR2* inhibit the proliferation, migration, and invasion of melanoma cells, such as SCH-527123 [33]. Although *CXCR2* participates in melanoma metastasis, only a few studies for *CXCR2P* are available. Whether *CXCR2P* participates in this process and the extent or mechanism of its participation are still unknown. Thus, further study is needed in the future.

The functions of the candidate genes are relevant to the immune system and inflammation and are consistent with the primary enrichment functions of upregulated DEGs. Melanoma microenvironment is composed of diverse immune cells and stromal cells that regulate the initiation and the development of disease and cellular response to therapies [34]. Innate immune cells in cutaneous melanoma, such as macrophages, NK cells, and dendritic cells, have strong plasticity and play a protumor or antitumor role through cell-to-cell and cell-to-tumor interactions and soluble molecules in the microenvironment [35]. This finding could explain why some genes are bidirectional in regulating tumors. Hence, this bidirectional action should be considered and explored from multiple perspectives when discussing the relationship between genes and melanoma. Tumor-related inflammation caused by the accumulation of white blood cells, which secrete cytokines and chemokines, can actively remodel tissues and angiogenesis, leading to conditions conducive to tumor growth, invasion, and metastasis [36]. However, the relationship between candidate genes and cutaneous metastatic melanoma is still unclear and warrants additional research.

In the clinical analysis, *MZB1* has the biggest effect on the overall survival time. Furthermore, the rate of lymph node metastasis is higher in the *MZB1* high expression group than in the low expression group. This result coincides with other studies that *MZB1* regulates lymphocyte adhesion and migration. The number of patients exhibiting a tumor-free status was higher in the *MZB1* high expression group than in the low expression group. However, the patients’ economic status, basic diseases, surgical approach, or other treatments, all of which can influence clinical outcomes, were not considered.

Cutaneous metastatic melanoma always has a worse prognosis than primary melanoma. Patients with advance-staged melanoma do not respond well to the treatment due to primary or acquired resistance. In addition, only a few studies on the genes related to the prognosis of metastatic melanoma have been performed. In the present study, we determined four candidate genes associated with metastatic melanoma that could be potentially prognostic risk factors for patients with cutaneous metastatic melanoma. We found that all of these genes are related to immunity and inflammation, but the specific processes of how these genes participated in metastatic melanoma have not been proven. This study provides a new direction for further research on metastatic melanoma and may provide a new target for the treatment or prevention of metastatic melanoma.
